# Using Movement to Regulate Emotion: Neurophysiological Findings and Their Application in Psychotherapy

**DOI:** 10.3389/fpsyg.2016.01451

**Published:** 2016-09-23

**Authors:** Tal Shafir

**Affiliations:** ^1^The Graduate School of Creative Arts Therapies, Faculty of Social Welfare and Health Sciences, University of HaifaHaifa, Israel; ^2^The Department of Psychiatry, University of Michigan, Ann ArborMI, USA

**Keywords:** emotion regulation, dance/movement therapy, psychotherapy, embodiment, embodied simulation, empathy, neuroscience, motor behavior

## Abstract

Emotion regulation is a person’s active attempt to manage their emotional state by enhancing or decreasing specific feelings. Peripheral theories of emotion argue that the origins of emotions stem from bodily responses. This notion has been reformulated in neurophysiological terms by Damasio, who claimed that emotions are generated by conveying the current state of the body to the brain through interoceptive and proprioceptive afferent input. The resulting brain activation patterns represent unconscious emotions and correlate with conscious feelings. This proposition implies that through deliberate control of motor behavior and its consequent proprioception and interoception, one could regulate his emotions and affect his feelings. This concept is used in dance/movement (psycho)therapy where, by guiding to move in a certain way, the therapist helps the client to evoke, process, and regulate specific emotions. Exploration and practice of new and unfamiliar motor patterns can help the client to experience new unaccustomed feelings. The idea that certain motor qualities enhance specific emotions is utilized by the therapist also when she mirrors the client’s movements or motor qualities in order to feel what the client feels, and empathize with them. Because of the mirror neurons, feeling what the client feels is enabled also through observation and imagination of their movements and posture. This principle can be used by verbal therapists as well, who should be aware of its bi-directionality: clients seeing the therapist’s motor behavior are unconsciously affected by the therapist’s bodily expressions. Additional implications for psychotherapy, of findings regarding mirror neurons activation, are discussed.

One of the goals often sought out in psychotherapy is emotion regulation. Emotion regulation is defined as a person’s active attempt to manage his emotional state by enhancing or decreasing specific feelings. This is the “processes by which individuals influence which emotions they have, when they have them, and how they experience and express these emotions” (Gross, 1998, 2002). Emotion regulation is essential for healthy psychological functioning. Deficits in the regulation of interpersonal emotions have been linked to psychiatric disorders, and teaching patients emotion regulation strategies has improved symptoms in a variety of emotional disorders (Farchione et al., 2012). While most previous studies of applied techniques for emotion regulation concentrated on cognitive strategies such as reappraisal, distancing, and distraction, or on behavioral strategies such as exposure for desensitization or response modulation, one of the most readily available but underutilized strategies is emotion regulation through changes to posture and movement. Until recent years this strategy was considered a body-mind based alternative therapy approach and was rarely studied scientifically, but it is in fact highly compatible with neurophysiological findings.

Peripheral theories of emotion argue that the origin of emotional feelings stem from bodily responses. This notion, suggested by Darwin (1872) and James (1884), has been reformu- lated in neurophysiological terms by Damasio. According to Damasio, emotions are generated by conveying the current state of the body to the brain through interoception (input representing the physiological state of the body, such as thermal, metabolic, hormonal) and proprioception (input from muscles and joints). The resulting brain activation patterns represent unconscious emotions, and correlate with subjective conscious feelings (Damasio, 1999; Damasio et al., 2000; Damasio and Carvalho, 2013). Damasio’s framework implies that through deliberate control of motor behavior and its consequent proprioception and interoception, one could regulate one’s feelings (Riskind, 1984).

Another neurophysiological finding supporting emotion regulation through movement, is the mirror neurons activation during motor observation. Although the linkage between mirror neuron activity and the behavior of larger neuronal networks is unknown, it was suggested that mirror neurons have a role in embodied simulation. It is posited that embodied simulation creates in the observer an internal simulation of the observed movements, leading to elicitation of the same emotions felt and expressed by the observed moving person (Niedenthal, 2007; Heberlein and Atkinson, 2009; Gallese and Sinigaglia, 2011). This embodied simulation process that takes place during motor observation is the base for emotional empathy (Nummenmaa et al., 2008; Decety, 2011).

Lastly, it was found that during motor imagery, the motor system is activated similar to its activation during motor execution (Grezes and Decety, 2001; Filimon et al., 2007), except for an additional inhibition of the final motor output (Guillot et al., 2012), leading to embodied simulation of the movements. This in turn generates a simulated sensory experience of those movements (Naito et al., 2002) and sometimes even real changes in heart rate and respiration (Decety et al., 1991) that consequently can elicit the associated emotion, similar to its elicitation by motor execution of the same movements. The following paragraphs will demonstrate how the regulatory effects on emotions, of motor execution, observation and imagery, are utilized in psychotherapy.

Different types of motor-behavior modifications contribute to emotion regulation based on different underlying mechanisms (Shafir, 2015). *Quantitative* changes in motor behavior, i.e., increased intensity and/or duration of muscular activity such as during exercise, produce changes in autonomic nervous system activation (e.g., increased heart rate) and in metabolic processes, which generate a myriad of physiological changes (e.g., alterations in the levels of hormones, neurotransmitters, trophic factors, endocannabinoids, immune system function) that both elevate mood and contribute to the reduction of stress, anxiety, and depression. In addition, there is evidence to suggest that *qualitative* modifications of motor behavior, such as engaging in specific facial expressions, postures, and whole body movements, probably use a different mechanism to enhance their corresponding affects. This mechanism is likely based on proprioceptive input to the brain regarding the current state of the body’s muscle activation pattern and joint configuration, and existing associations in the brain between certain proprioceptive input and specific emotions (Lee et al., 2006; Hennenlotter et al., 2009). These associations are probably partly innate and partly learned: some associations, such as between freezing and fear, between shrinking and lowering the body and expression of timidity and submissiveness, or between displacement activities such as grooming or lip biting and stress (Troisi, 2002), exist also in the animal kingdom and are probably innate. Other associations are probably learned through the process of Hebbian learning, similar to the learning process of conceptual representations of actions, based on the theory of embodied semantics for actions (Aziz-Zadeh and Damasio, 2008), although this proposition has yet to be proven by research. Two other muscle-activation based strategies for emotion regulation are progressive muscle relaxation, which reduces stress (Pawlow and Jones, 2005), and specific breathing patterns, which are capable of reducing stress (Brown and Gerbarg, 2005) or inducing differentiated emotional states (Philippot et al., 2002).

Exercise (i.e., quantitative changes in motor behavior) has been shown to be effective in reducing mild to moderate depression in both clinical and non-clinical populations (Rethorst et al., 2009; Josefsson et al., 2014) as well as reducing anxiety in a variety of anxiety disorders (Strohle, 2009; Asmundson et al., 2013). As a result, exercise is now increasingly recommended as a natural, safer, low-cost alternative to medication, or as an augmented intervention alongside medication, for a series of mental conditions and disorders. Progressive muscle relaxation and specific breathing patterns are often taught and used for stress reduction as part of the strategies taught in cognitive behavioral therapy. As for the associations between certain postures and movements and specific emotions, they are widely used in somatic therapies and in dance/movement (psycho)therapy (DMT). These associations between specific movements and corresponding emotions are used in DMT for regulation (i.e., elicitation or diminution) of specific emotions through motor execution, motor observation, and motor imagery of their associated movements, and all three of these processes can be used in the practice of other forms of psychotherapy as well.

During motor execution, as mentioned above, the proprio- ceptive input from the muscles and joints to the brain evokes an associated emotion. Since our body is always in some type of a posture, whether we lie down, sit, stand or are in motion, the posture that an individual assumes and the type of movements he is engaged in have a constant and continuous effect on his affective state. Different postures and movement patterns are associated with and evoke different emotions (Duclos and Laird, 2001; Carney et al., 2010; Shafir et al., 2013, 2016; Koch, 2014; Koch et al., 2014). This concept is used in behavioral therapy, when patients are encouraged to smile as a behavioral intervention to help them elevate their mood, even when the smile is initially artificial. The activation of the facial muscles into an expression that is associated with happiness evokes or enhances this associated emotion, leading to the improvement in mood. This notion is similarly used in DMT in various ways, by activating muscles of the entire body: to help clients bring up and/or process their feelings, dance/movement therapists guide clients to move their entire bodies in particular ways.

Clients are often encouraged during a DMT session to embody, improvise and express in movement the problem they are trying to solve and how they would go about solving it, as well as their attitude toward a certain person or situation, or their behavioral or emotional response to specific conditions or stimuli (Bernstein, 1995). These movements elicit and enhance the emotions that are associated with them, helping the client to consciously identify, fine-tune and process the associated feelings (Mills and Daniluk, 2002). In addition, therapists help clients to explore their feelings by suggestions to move in ways associated with different attitudes (Ginsburgs and Goodill, 2009). Therapists also guide clients to regulate their emotions by suggesting to move in specific ways which promote movements associated with a desired emotion, and/or by suggesting to reduce and avoid motor patterns associated with undesired emotions. For example, by suggesting to practice moving with the head erect, the gaze directed straight forward, and with his back straight and chest raised and expanded, a therapist can help a client to experience and increase feelings of self-confidence and pride. Conversely, giving “homework” to a client, to consciously avoid looking down, slumping his shoulders and chest, etc., during daily activities, can help reduce feeling “down.” Another important way by which dance/movement therapists use movements to affect their clients’ emotional state is inviting the client to explore and practice new and unfamiliar motor patterns. By guiding the client to expand their motor vocabulary, the therapist helps them to learn and practice new motor patterns, leading to experience of desirable feelings that the client may not have had access to before.

The principle of motor execution as a mean to affect emotion can be used not only in DMT but also in other forms of psychotherapy by, for example, asking clients to change their sitting posture during a verbal therapy session: if a client who is used to sitting in a closed, bent posture changes his sitting posture into an open, erect one, this could affect his entire experience during the therapy session.

The principle of motor execution as a way to enhance the associated feelings can be utilized not only by clients, but by therapists as well. It is not uncommon for a DMT therapist to mirror her client’s movements or motor qualities. While motor mirroring usually serves to give feedback to the client and/or a feeling of being noticed and accepted, it is also used to increase the therapist’s empathy and understanding of the client’s emotional state (McGarry and Russo, 2011). Imitating the client’s movements or the quality of their movements evokes in the therapist the same emotions that are felt by the moving client. This too can be done by a verbal psychotherapist. By mirroring the sitting posture of a client and the changes in his posture throughout the therapeutic session, the therapist can better feel the client’s emotional fluctuations during the session.

In addition to motor execution, motor observation can also enhance in the observer the emotion associated with the observed movements (Shafir et al., 2013). This is accomplished probably through activation of the mirror neurons, as explained above. By observing the client’s movements while paying special attention to the feelings they evoke within her, the therapist can better feel what the client is feeling. This is true not only for dance/movement therapists but for any (verbal or other) therapist observing clients’ movements both as they enter the room and during the therapy session. Movements for this purpose need not only be big movements involving the entire body, as are often performed during DMT sessions, but can be any type of movement performed while sitting and talking, including facial expressions, gestures, displacement activities that express stress (Troisi, 2002), changing sitting positions, etc.

It should be mentioned, however, that although motor observation enhances the feelings associated with the observed movements, research has shown that motor execution leads to more intense feelings when compared to the feelings evoked by motor observation of the same movements (Shafir et al., 2013). Thus, if a therapist observing a client is unsure about the client’s feelings, mimicking the client or moving with similar motor qualities (Shafir et al., 2016) could help the therapist clarify and identify those feelings, by enhancing them within herself. Indeed, Sletvold (2015) adopted this idea in his model for clinical supervision, in which the supervisee is asked to assume physically the position of his patient and from there to explore embodied empathy in depth.

Although mirroring the client’s movements is a technique often used in DMT, it is sometimes inappropriate, and the therapist must rely solely on motor observation to understand and empathize with the client’s emotions. On such occasions, it is important to know that the ability to transform observations of motor expressions into an internal simulation of those movements and thus feel the associated emotions is a skill that therapists can develop and improve. Two neuroscientific findings support this notion. The first is Catmur et al.’s (2007, 2008) finding that the mirror neuron system is plastic and develops through sensorimotor learning. The implication of this finding is that by practicing unfamiliar movements which are not normally within a therapist’s motor repertoire, such as for example, stereotypical movements of autistic children, dyskinetic movements of people with Parkinson, or very fast, frantic movements for a therapist whose natural motor behavior is slow and calm, dance/movement therapists can teach themselves to better sense the feelings associated with and evoked by such unfamiliar movements. The second discovery is that of Calvo-Merino et al. (2005, 2006) who found that the mirror system is activated more when we observe movements that are within our motor repertoire, as compared to movements that we have little or no experience doing. This finding implies that the more personal experience one has moving a certain movement, the easier it will probably be for her to internally simulate that movement and feel its associated emotion. This finding emphasizes the importance for dance/movement therapists of having a wide range of movement experiences and large motor vocabulary: the more experience a dance/movement therapist has in a variety of different movement styles, the easier it will be for her to mirror, empathize and “feel” a variety of people moving with diverse motor patterns.

One important thing to remember in relation to the activation of the mirror neurons during motor observation is that it happens to everyone. This means that, similar to the ability of the therapist to infer what the client is feeling based on the client’s movements and posture, the client can feel the therapist’s feelings when observing the therapist’s body language. Therapists should be aware of this reciprocity and constantly monitor and control their body language, avoiding movements and postures that can negatively affect clients, and adopting postures and movements that can positively affect the therapeutic process. We all have a natural tendency to automatically and unconsciously mimic the behavior of the people we interact with (Chartrand and Lakin, 2013). During a therapeutic session with for example, a depressed client, the therapist should be aware of how much she automatically adopts the hopeless, lethargic posture of the client in front of her. While adopting a slightly similar posture may give the client a sense of empathy, fully adopting the client’s helpless, slumped posture might give the client a message of despair and the inability of the therapist to help him. Another obvious example would be to consciously avoid any facial or bodily expression of disgust that a client may evoke in a therapist.

Although some clients may feel uncomfortable seeing themselves being mirrored, studies have shown that most people feel more positive about someone who imitates them. While some researchers ascribed this phenomenon to increased social bonding and interpersonal closeness (Lakin et al., 2003) or an indication of pre-existing rapport (Scheflen, 1964), recent findings suggest that the reason is that observing mimicry triggers reward related processing regions in the brain, leading to elicitation of positive affect. Kühn et al. (2010) has shown that being imitated compared to not being imitated activates brain areas that have been associated with emotion and reward processing, namely the medial orbitofrontal cortex/ventromedial prefrontal cortex, and that these regions show higher effective connectivity with the striatum and mid posterior insula while being imitated compared to not being imitated. This phenomenon is used in DMT when the therapist joins the client and moves in front of him or at his side, mirroring his movements. Such behavior of the therapist often makes the client feel reassured, supported and empathized by the therapist, and it strengthens the connection between client and therapist (McGarry and Russo, 2011). This strategy too, can be utilized by other types of therapists, who can consciously imitate their clients’ small gestures or changes in sitting position during therapeutic sessions. In fact, extant literature contains several examples that demonstrate the effectiveness of such a strategy: Maurer and Tindall (1983) found that high school juniors perceived their counselors’ level of empathy as higher when the counselors sat in a posture that was congruent with their own posture during the counseling session when compared to a non-congruent posture. Ramseyer and Tschacher (2011) found that psychotherapeutic relationships that were characterized by higher non-verbal synchrony between the patient and therapist during face to face sessions were rated by the patients as having higher relationship quality between the patient and therapist, and they increased the patients’ self-efficacy more than therapeutic relationships characterized by lower such non-verbal synchrony. In addition, patients with high synchrony had a higher reduction of symptoms and less insecure attachment patterns at the end of treatment.

Motor imagery has also been found to enhance the emotions associated with imagined movements (Shafir et al., 2013). Motorically disabled or physically sick clients with limited motor capabilities can use motor imagery instead of motor execution to elicit desired emotions, imagining themselves, for example, running on the beach or dancing at a party, to improve their mood. Clients can also use motor imagery as part of practicing through mental simulation a desired behavioral response, for example, by imagining themselves standing in a grounded, open, and firm posture, to help themselves feel more confident and self-assured when mentally practicing being assertive during a difficult social interaction. Therapists, on the other hand, could use motor imagery to enhance the empathic effects of motor observation, by imagining themselves doing the movements they observe their clients doing.

In conclusion, based on peripheral theories of emotion, the mirror neuron system, and specific brain activation during motor observation and motor imagery, motor behavior, its observation, and its imagination can affect one’s emotional state, and these can be utilized in various ways during psychotherapy.

## Author Contributions

The author confirms being the sole contributor of this work and approved it for publication.

## Conflict of Interest Statement

The author declares that the research was conducted in the absence of any commercial or financial relationships that could be construed as a potential conflict of interest.

## References

[B1] Asmundson G. J. G. Fetzner M. G. DeBoer L. B. Powers M. B. Otto M. W. Smits J. A. J. (2013). Lett’s get physical: a contemporary review of the anxiolytic effects of exercise for anxiety and its disorders. *Depress. Anxiety* 30 362–373. 10.1002/da.22043 23300122

[B2] Aziz-Zadeh L. Damasio A. (2008). Embodied semantics for actions: findings from functional brain imaging. *J. Physiol.Paris* 102 35–39. 10.1016/j.jphysparis.2008.03.012 18472250

[B3] Bernstein B. (1995). “Dancing beyond Trauma: women survivors of sexual abuse,” in *Dance and Other Expressive Art Therapies: When Words Are Not Enough* ed. Levy F. (New York, NY: Routledge) 41–58.

[B4] Brown R. P. Gerbarg P. L. (2005). Sudarshan Kriya yogic breathing in the treatment of stress, anxiety, and depression: part I-neurophysiologic model. *J. Altern. Complement. Med.* 11 189–201. 10.1089/acm.2005.11.189 15750381

[B5] Calvo-Merino B. Glaser D. E. Grezes J. Passingham R. E. Haggard P. (2005). Action observation and acquired motor skills: an fMRI study with expert dancers. *Cereb. Cortex* 15 1243–1249. 10.1093/cercor/bhi007 15616133

[B6] Calvo-Merino B. Grèzes J. Glaser D. E. Passingham R. E. Haggard P. (2006). Seeing or doing? influence of visual and motor familiarity in action observation. *Curr. Biol.* 16 1905–1910. 17027486 10.1016/j.cub.2006.07.065

[B7] Carney D. R. Cuddy A. J. C. Yap A. J. (2010). Power posing brief nonverbal displays affect neuroendocrine levels and risk tolerance. *Psychol. Sci.* 21 1363–1368. 10.1177/0956797610383437 20855902

[B8] Catmur C. Gillmeister H. Bird G. Liepelt R. Brass M. Heyes C. (2008). Through the looking glass: counter-mirror activation following incompatible sensorimotor learning. *Eur. J. Neurosci.* 28 1208–1215. 10.1111/j.1460-9568.2008.06419.x 18783371

[B9] Catmur C. Walsh V. Heyes C. (2007). Sensorimotor learning configures the human mirror system. *Curr. Biol.* 17 1527–1531. 10.1016/j.cub.2007.08.006 17716898

[B10] Chartrand T. L. Lakin J. L. (2013). The antecedents and consequences of human behavioral mimicry. *Annu. Rev. Psychol.* 64 285–308. 10.1146/annurev-psych-113011-143754 23020640

[B11] Damasio A. Carvalho G. B. (2013). The nature of feelings: evolutionary and neurobiological origins. *Nat. Rev. Neurosci.* 14 143–152. 10.1038/nrn3403 23329161

[B12] Damasio A. R. (1999). *The Feeling of What Happens : Body and Emotion in the Making of Consciousness*. New York, NY: Harcourt Brace.

[B13] Damasio A. R. Grabowski T. J. Bechara A. Damasio H. Ponto L. L. B. Parvizi J. (2000). Subcortical and cortical brain activity during the feeling of self-generated emotions. *Nat. Neurosci.* 3 1049–1056. 10.1038/79871 11017179

[B14] Darwin C. R. (1872). *The Expression of the Emotions in Man and Animals.* London: John Murray.

[B15] Decety J. (2011). Dissecting the neural mechanisms mediating empathy. *Emot. Rev.* 3 92–108. 10.1177/1754073910374662

[B16] Decety J. Jeannerod M. Germain M. Pastene J. (1991). Vegetative response during imagined movement is proportional to mental effort. *Behav. Brain Res.* 42 1–5. 10.1016/S0166-4328(05)80033-6 2029340

[B17] Duclos S. E. Laird J. D. (2001). The deliberate control of emotional experience through control of expressions. *Cogn. Emot.* 15 27–56. 10.1080/0269993004200088

[B18] Farchione T. J. Fairholme C. P. Ellard K. K. Boisseau C. L. Thompson-Hollands J. Carl J. R. (2012). Unified protocol for transdiagnostic treatment of emotional disorders: a randomized controlled trial. *Behav. Ther.* 43 666–678. 10.1016/j.beth.2012.01.001 22697453 PMC3383087

[B19] Filimon F. Nelson J. D. Hagler D. J. Sereno M. I. (2007). Human cortical representations for reaching: mirror neurons for execution, observation, and imagery. *Neuroimage* 37 1315–1328. 10.1016/j.neuroimage.2007.06.008 17689268 PMC2045689

[B20] Gallese V. Sinigaglia C. (2011). What is so special about embodied simulation? *Trends Cogn. Sci.* 15 512–519. 10.1016/j.tics.2011.09.003 21983148

[B21] Ginsburgs V. Goodill S. (2009). A dance/movement therapy clinical model for women with gynecologic cancer undergoing high dose rate brachytherapy. *Am. J. Dance Ther.* 31 136–158. 10.1007/s10465-009-9076-0

[B22] Grezes J. Decety J. (2001). Functional anatomy of execution, mental simulation, observation, and verb generation of actions: a meta-analysis. *Hum. Brain Mapp.* 12 1–19. 10.1002/1097-0193(200101)12:1<1::AID-HBM10>3.0.CO;2-V 11198101 PMC6872039

[B23] Gross J. J. (1998). The emerging field of emotion regulation: an integrative review. *Rev. Gen. Psychol.* 2 271–299. 10.1037/1089-2680.2.3.271

[B24] Gross J. J. (2002). Emotion regulation: affective, cognitive, and social consequences. *Psychophysiology* 39 281–291. 10.1017/S0048577201393198 12212647

[B25] Guillot A. Di Rienzo F. MacIntyre T. Moran A. P. Collet C. (2012). Imagining is not doing but involves specific motor commands: a review of experimental data related to motor inhibition. *Front. Hum. Neurosci.* 6:247 10.3389/fnhum.2012.00247 PMC3433680 22973214

[B26] Heberlein A. S. Atkinson A. P. (2009). Neuroscientific evidence for simulation and shared substrates in emotion recognition: beyond faces. *Emot. Rev.* 1 162–177. 10.1177/1754073908100441

[B27] Hennenlotter A. Dresel C. Castrop F. Ceballos-Baumann A. O. Wohlschlager A. M. Haslinger B. (2009). The link between facial feedback and neural activity within central circuitries of emotion–new insights from botulinum toxin-induced denervation of frown muscles. *Cereb. Cortex* 19 537–542. 10.1093/cercor/bhn104 18562330

[B28] James W. (1884). What is emotion. *Mind* 9 188–205. 10.1093/mind/os-IX.34.188

[B29] Josefsson T. Lindwall M. Archer T. (2014). Physical exercise intervention in depressive disorders: Meta-analysis and systematic review. *Scand. J. Med. Sci. Sports* 24 259–272. 10.1111/sms.12050 23362828

[B30] Koch S. C. (2014). Rhythm is it: effects of dynamic body feedback on affect and attitudes. *Front. Psychol.* 5:537 10.3389/fpsyg.2014.00537 PMC4051267 24959153

[B31] Koch S. C. Fuchs T. Summa M. (2014). Body memory and kinesthetic body feedback: the impact of light versus strong movement qualities on affect and cognition. *Mem. Stud.* 7 272–284. 10.1177/1750698014530618

[B32] Kühn S. Müller B. C. van Baaren R. B. Wietzker A. Dijksterhuis A. Brass M. (2010). Why do I like you when you behave like me? Neural mechanisms mediating positive consequences of observing someone being imitated. *Soc. Neurosci.* 5 384–392. 10.1080/17470911003633750 20229392

[B33] Lakin J. L. Jefferis V. E. Cheng C. M. Chartrand T. L. (2003). The chameleon effect as social glue: evidence for the evolutionary significance of nonconscious mimicry. *J. Nonverbal Behav.* 27 145–162. 10.1023/A:1025389814290

[B34] Lee T.-W. Josephs O. Dolan R. J. Critchley H. D. (2006). Imitating expressions: emotion-specific neural substrates in facial mimicry. *Soc. Cogn. Affect. Neurosci.* 1 122–135. 10.1093/scan/nsl012 17356686 PMC1820946

[B35] Maurer R. E. Tindall J. H. (1983). Effect of postural congruence on client’s perception of counselor empathy. *J. Couns. Psychol.* 30 158–163. 10.1037/0022-0167.30.2.158

[B36] McGarry L. M. Russo F. A. (2011). Mirroring in dance/movement therapy: potential mechanisms behind empathy enhancement. *Arts Psychother.* 38 178–184. 10.1016/j.aip.2011.04.005

[B37] Mills L. J. Daniluk J. C. (2002). Her body speaks: the experience of dance therapy for women survivors of child sexual abuse. *J. Couns. Dev.* 80 77–85. 10.1002/j.1556-6678.2002.tb00169.x

[B38] Naito E. Kochiyama T. Kitada R. Nakamura S. Matsumura M. Yonekura Y. (2002). Internally simulated movement sensations during motor imagery activate cortical motor areas and the cerebellum. *J. Neurosci.* 22 3683–3691. 11978844 10.1523/JNEUROSCI.22-09-03683.2002 PMC6758350

[B39] Niedenthal P. M. (2007). Embodying emotion. *Science* 316 1002–1005. 10.1126/science.1136930 17510358

[B40] Nummenmaa L. Hirvonen J. Parkkola R. Hietanen J. K. (2008). Is emotional contagion special? An fMRI study on neural systems for affective and cognitive empathy. *Neuroimage* 43 571–580. 10.1016/j.neuroimage.2008.08.014 18790065

[B41] Pawlow L. A. Jones G. E. (2005). The impact of abbreviated progressive muscle relaxation on salivary cortisol and salivary immunoglobulin A (sIgA). *Appl. Psychophysiol. Biofeedback* 30 375–387. 10.1007/s10484-005-8423-2 16385425

[B42] Philippot P. Chapelle G. Blairy S. (2002). Respiratory feedback in the generation of emotion. *Cogn. Emot.* 16 605–627. 10.1080/02699930143000392

[B43] Ramseyer F. Tschacher W. (2011). Nonverbal synchrony in psychotherapy: coordinated body movement reflects relationship quality and outcome. *J. Consult. Clin. Psychol.* 79 284–295. 10.1037/a0023419 21639608

[B44] Rethorst C. Wipfli B. Landers D. (2009). The antidepressive effects of exercise. *Sports Med.* 39 491–511. 10.2165/00007256-200939060-00004 19453207

[B45] Riskind J. H. (1984). They stoop to conquer: guiding and self-regulatory functions of physical posture after success and failure. *J. Pers. Soc. Psychol.* 47 479–493. 10.1037/0022-3514.47.3.479

[B46] Scheflen A. E. (1964). The significance of posture in communication systems. *Psychiatry* 27 316–331. 14216879 10.1080/00332747.1964.11023403

[B47] Shafir T. (2015). “Movement-based strategies for emotion regulation,” in *Handbook on Emotion Regulation: Processes, Cognitive Effects and Social Consequences* ed. Bryant M. L. (New York, NY: Nova Science Publishers, Inc) 231–249.

[B48] Shafir T. Taylor S. F. Atkinson A. P. Langenecker S. A. Zubieta J.-K. (2013). Emotion regulation through execution, observation, and imagery of emotional movements. *Brain Cogn.* 82 219–227. 10.1016/j.bandc.2013.03.001 23561915 PMC4067484

[B49] Shafir T. Tsachor R. P. Welch K. (2016). Emotion regulation through movement: unique sets of movement characteristics are associated with and enhance basic emotions. *Front. Psychol.* 6:2030 10.3389/fpsyg.2015.02030 PMC4707271 26793147

[B50] Sletvold J. (2015). Embodied empathy in psychotherapy: Demonstrated in supervision. *Body Mov. Dance Psychother.* 10 82–93. 10.1080/17432979.2014.971873

[B51] Strohle A. (2009). Physical activity, exercise, depression and anxiety disorders. *J. Neural. Transm.* 116 777–784. 10.1007/s00702-008-0092-x 18726137

[B52] Troisi A. (2002). Displacement activities as a behavioral measure of stress in nonhuman primates and human subjects. *Stress* 5 47–54. 10.1080/102538902900012378 12171766

